# *PSCA* rs2294008 polymorphism contributes to the decreased risk for cervical cancer in a Chinese population

**DOI:** 10.1038/srep23465

**Published:** 2016-03-22

**Authors:** Shizhi Wang, Shenshen Wu, Haixia Zhu, Bo Ding, Yunlang Cai, Jing Ni, Qiang Wu, Qingtao Meng, Xin Zhang, Chengcheng Zhang, Xiaobo Li, Meilin Wang, Rui Chen, Hua Jin, Zhengdong Zhang

**Affiliations:** 1 Key Laboratory of Environmental Medicine Engineering, Ministry of Education, School of Public Health, Southeast University, Nanjing, China; 2 Core Laboratory, Nantong Tumor Hospital, Nantong, China; 3 Department of Gynecology and Obstetrics, Zhongda Hospital, School of Medicine, Southeast University, Nanjing, China; 4 Department of Gynecologic Oncology, Affiliated Cancer Hospital of Nanjing Medical University, Jiangsu Cancer Hospital, Nanjing, China; 5 Department of Environmental Genomics, Jiangsu Key Laboratory of Cancer Biomarkers, Prevention and Treatment, Cancer Center, Nanjing Medical University, Nanjing, China; 6 Department of Genetic Toxicology, the Key Laboratory of Modern Toxicology of Ministry of Education, School of Public Health, Nanjing Medical University, Nanjing, China

## Abstract

Recently, three genome-wide association studies have identified the *PSCA* (prostate stem cell antigen) rs2294008 polymorphism (C > T) associated with susceptibility to gastric cancer, bladder cancer, and duodenal ulcers, highlighting its critical role in disease pathogenesis. Given PSCA is reported to be overexpressed in cervical cancer and the rs2294008 can influence *PSCA* transcription, we aimed to determine the role of rs2294008 in susceptibility to cervical cancer. The genotyping was performed in the 1126 cases and 1237 controls. Our results showed the rs2294008 TT genotype significantly associated with a reduced risk of cervical cancer (adjusted OR = 0.55, 95% CI = 0.38–0.79; recessive model). Stratified analyses revealed that the association was restricted to the subgroups of age > 49 years, parity ≤ 1, abortion and early-stage cervical cancer. Immunohistochemistry assay showed the individuals carrying the TT genotype having lower PSCA expression than those with CC/CT genotypes. In summary, the *PSCA* rs2294008 polymorphism may serve as a biomarker of cervical cancer, particularly of early-stage cervical cancer.

Genome-wide association studies (GWAS) are non-candidate gene-driven and use a whole-genome approach in large studies of up to hundreds of thousands of individuals investigating traits such as disease, response to drug and anthropometry^1^. GWAS are possible to overcome the constraints of candidate-gene driven studies and identify novel disease-related genes because they investigate all human genes in the whole genome instead of specific genes or chromosome regions^2^. Recently, three genome-wide association studies (GWAS) performed in Asian and Caucasian populations have revealed the association between the *PSCA* (prostate stem cell antigen) rs2294008 polymorphism and susceptibility to gastric cancer^3^, bladder cancer^4^, and duodenal ulcers^5^. These results implicated a critical role of *PSCA* and its genetic variant in the etiology of multiple complex diseases. PSCA is a 123-amino-acid glycoprotein related to the Ly-6 family of cell-surface protein, and first identified in the LAPC-4 prostate xenograft model of human prostate cancer in 1998^6^. PSCA was initially considered as the prostate-specific cell-surface antigen, but its expression has subsequently exhibited in extraprostatic normal tissues, such as esophagus, stomach, bladder, and cervix^7^,^8^.

It has been reported that PSCA is involved in the signal transduction and cell-growth regulation^3^,^9^, however, the precise role of PSCA remains unknown and controversial. Apart from prostate cancer, overexpressed PSCA has also been found in a proportion of non-prostatic malignancies, including cervical^8^, bladder^10^, and pancreatic^11^ cancers, suggesting an oncogenic role in tumor development. Marra *et al*.^9^ reported that down regulation of the PSCA expression was associated with reduced cell proliferation of bladder cancer *in vitro* and *in vivo*, and moreover with activation of genes of downstream of the IFNα/β receptor. In contrast, reduced PSCA expression is observed in gastric^3^, gallbladder^12^, and head-and-neck squamous cell cancers^13^. Sakamoto *et al*.^3^ found that PSCA participated in gastric cell-proliferation inhibition and cell death activation, acting as a tumor suppressor gene. The discrepancy reflects the complexity of PSCA regulation, involving pro-tumorigenic and anti-tumorigenic functions in various contexts^14^. As to the possible role of PSCA in cervical cancer, Liu *et al*.^8^ reported that PSCA was highly expressed in cervical cancer than in adjacent normal cervix, and positively correlated with invasion of cervical cancer, implying an oncogenic role in cervical carcinogenesis.

The SNP rs2294008 polymorphism (C > T, Met1Thr), located in the translation starting site of *PSCA* gene, can lead to an alternative splice variant of *PSCA* with an additional fragment of 9 amino acids at the N-terminal portion and change protein localization from cytoplasm to cell surface^3^. After the first reported by Sakamoto *et al*.^3^, some other studies performed in China^15^,^16^,^17^, Japan^18^ and European populations^19^,^20^ have validated the link between the polymorphism and gastric cancer risk. Besides gastric cancer, two other GWAS have identified its association with bladder cancer^4^ and duodenal ulcers^5^ susceptibility in US and Japan populations, respectively. The SNP has also shown to correlate with susceptibility to other solid tumors, including esophagus^19^ and breast cancers^21^. Taking into account the potential oncogenic role of PSCA in cervical cancer and the attractive statistical significance of the rs2294008 polymorphism among the three GWAS, we aimed to determine the association of the rs2294008 polymorphism with risk of cervical cancer in a Chinese population.

## Materials and Methods

### Ethics statement

The study was approved by the institutional review board of Southeast University. Each subject signed an informed consent. The research protocol was carried out in accordance with the approved guidelines.

### Study subjects

The study enrolled two independent sets of subjects. The test set comprised of 571 case patients and 657 healthy controls recruited from hospitals in Nanjing and Wuxi cities between January 2007 and December 2010. The validation set comprised of 555 case and 580 controls recruited from hospitals in Nantong City between January 2009 and December 2013. All patients were newly diagnosed with histologically confirmed cervical cancer. Those who had metastasized cancers from other origin or preoperative chemo/radio-therapy were exclude. All controls were genetically unrelated to the cases and recruited from those who were seeking for health care in the same hospitals.

### Genotyping

Genomic DNA of the test set and controls of the validation set was extracted from the peripheral blood lymphocytes. The sections of formalin-fixed paraffin-embedded (FFPE) tissues were used for DNA extraction for the patients in the validation set, due to lack of blood samples. The genotypes of the rs2294008 polymorphism were determined by TaqMan allelic discrimination method equipped with ABI 7900HT Real Time PCR System (Applied Biosystems, CA, USA). Quality control for the genotyping was achieved by including in each amplification a negative PCR control sample and three positive control samples for each genotype. At least 10% of the samples were randomly selected for genotyping confirmation, and the results were 100% concordant.

### Immunohistochemistry (IHC)

IHC for PSCA was performed on representative paraffin sections of primary cervical cancer tissues. The procedure of immunostaining has been described in our previous publication^22^. Series of paraffin sections of specimens were incubated with polyclonal rabbit anti-PSCA antibody (BA1694, BOSTER, Wuhan, China) overnight at 4 °C, and 3,3′-diaminobenzidine (DAB; Zhongshan Biotech, Beijing, China) was used to produce a brown precipitate. The evaluation of PSCA expression was made in a blind fashion by two independent pathologists simultaneously. The final PSCA staining score was calculated by multiplying the intensity and the percentage of positive cells, and categorized as follows: ≤3, negative or weak; >3 but ≤6, moderate; and >6, strong.

### Statistical analysis

A goodness-of-fit chi-square test (χ^2^ test) was applied to test the Hardy-Weinberg equilibrium (HWE) of the controls’ genotype frequencies. Differences between cases and controls in demographic characteristics and frequencies of allele and genotype of rs2294008 were evaluated by *t* test (for continuous variables) or by χ^2^ test (for categorical variables). The magnitude of the association of rs2294008 variants in each group was estimated using odds ratios (ORs) and 95% confidence intervals (CIs). For all tests, a two-sided *P*-value < 0.05 was considered statistically significant.

## Results

### Demographic and clinical characteristics of the study subjects

The demographic and clinical characteristics of the cases and controls are shown in Table 1. There were no differences in the distribution of age and abortion between the cases and controls in both test and validation sets (age, *P* = 0.726 and 0.798, respectively; abortion, *P* = 0.565 and 0.942, respectively). More subjects with higher parity (≥2) were observed in the cervical cancer patients than in the controls in both test (41.7% vs. 24.8%) and validation (45.4% vs. 17.6) sets (all *P* < 0.001). More subjects with postmenopausal status were observed in the cervical cancer patients than in the controls in the test set (44.0% vs. 21.1%), whereas more subjects with premenopausal status were observed in the cervical cancer patients in the validation set (52.8% vs. 42.6%) (all *P* < 0.001). The proportion of patients with T1 stage in the validation set was similar with that in the test set (68% vs. 65%). The patients with T2 were more in the validation set than in the test set (34.4% vs. 25.8%), whereas the patients with T3 or T4 were less in the validation set than in the test set (0.6 vs. 6.2%).

### PSCA rs2294008 polymorphism and cervical cancer susceptibility

The genotype frequencies of rs2294008 polymorphism in the control group of both test and validation sets did not deviate significantly from those expected under Hardy-Weinberg equilibrium model (*P* = 0.672 and 0.105 for the test and validation set, respectively). We further combined the two sets for enlargement of statistic power, and found statistically significant differences in the distribution of the rs2294008 genotypes and alleles between the cases and controls (*P* = 0.002 and 0.005, respectively; Table 2). When the CC genotype used as the reference, the heterozygous genotype CT was not associated with risk of cervical cancer (adjusted OR = 0.91, 95% CI = 0.77–1.09), whereas the homozygous mutant TT genotype was associated with a significantly reduced risk of cervical cancer (adjusted OR = 0.53, 95% CI = 0.37–0.77). When a recessive genetic model was employed, the TT genotype conferred a predominantly decreased risk of cervical cancer whatsoever adjusting for confounder factors (adjusted OR = 0.55, 95% CI = 0.38–0.79; Table 2).

### Stratified analysis of PSCA rs2294008 polymorphism and cervical cancer risk

In the stratified analyses, the TT genotype conferred a predominantly decreased risk of cervical cancer in both test and validation sets (adjusted OR = 0.54, 95% CI = 0.33–0.89, and 0.56, 0.34–0.98 for the test and validation sets, respectively; Table 3). Further stratified analyses by demographic characteristics revealed that the association between the rs2294008 TT genotype and cervical cancer risk was predominant in both pre- and post- menopausal status (adjusted OR = 0.60, 95% CI = 0.37–0.97, and 0.50, 0.29–0.87 for premenopausal and postmenopausal status, respectively), but restricted to the subgroups of age >49 years, parity ≤ 1, and abortion (adjusted OR = 0.42, 95% CI = 0.25–0.72 for age >49 years; 0.63, 0.40–0.99 for parity ≤ 1; and 0.42, 0.26–0.69 for abortion; Table 3). We also evaluated the relationship between the rs2294008 variants and invasion of cervical cancer, and found that the polymorphism was significantly associated with early-stage cervical cancer (adjusted OR = 0.53, 95% CI = 0.34–0.81; Table 3).

### rs2294008 polymorphism and PSCA protein expression

We then evaluated the association between the rs2294008 and PSCA protein expression in 50 cervical cancer tissues by IHC assay. We first genotyped rs2294008 in a total of 100 cervical cancer patients which have additional paraffin sections for IHC staining. The frequency distribution of CC, CT, and TT was 55, 39, and 6, respectively. Because the CC genotype was the wild genotype carried by the most cases and the TT was the homozygous mutant genotype carried by the least cases, we chose 25 (a half of 50 cases) cases carrying the CC genotypes, and kept all 6 cases carrying the TT genotypes. Finally, we chose 19 cases carrying the CT genotypes, which making the ratio between the CC and CT/TT genotypes 1:1.

**Figure 1:**
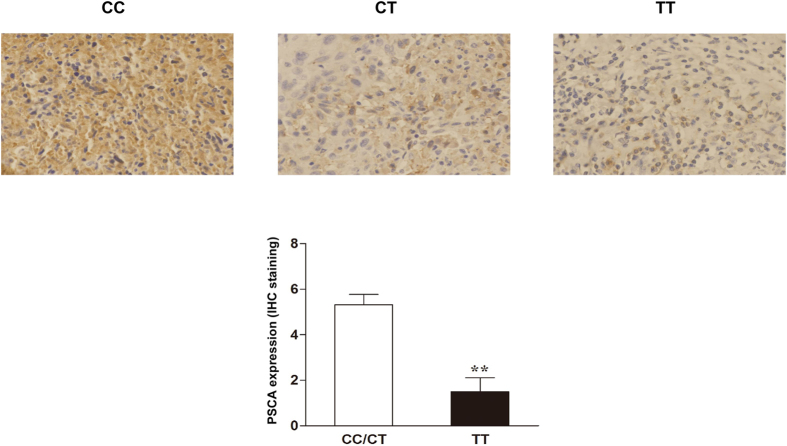
Immunohistochemical staining of PSCA in human cervical cancer tissues with different rs2294008 genotypes. Upper panel, representative images were obtained at 400× magnification. The frequency distribution of the CC, CT, and TT genotypes of rs2294008 was 25, 19, and 6, respectively. Bottom, the histogram of the PSCA expression in each genotype. **P < 0.01.

The representative IHC staining of PSCA in cervical cancer tissues with different rs2294008 genotypes was shown in Fig. 1 (upper panel). PSCA was expressed in the cytoplasm. There was a significant difference in cytoplasm staining of tissues between the patients with TT genotype and those with CC/CT genotypes (average staining score: 1.50 versus 5.32 for TT and CC/CT genotypes, respectively, *P* = 0.004; Fig. 1 bottom).

## Discussion

*PSCA* rs2294008 polymorphism has been identified by three GWAS in association with gastric cancer, bladder cancer and duodenal ulcer, highlighting its important role in disease pathogenesis. After its first reported by Sakamoto *et al*.^3^, several studies performed in Asian and Caucasian populations have validated its significant association with gastric and bladder cancer susceptibility. Furthermore, a number of studies have evaluated the relationship between this polymorphism and other solid tumors, including prostate, breast, colorectal, pancreatic, and esophageal tumors. To date, there is no report regarding the rs2294008 polymorphism in relation to susceptibility to cervical cancer. In the present study, we demonstrated that the homozygous variant TT genotype conferred a significantly reduced risk of cervical cancer compared with the wild-type containing genotypes. Stratified analysis showed that the association was restricted to the subgroups of no abortion and premenopausal status. In addition, the polymorphism correlated to the early-stage cervical cancer.

PSCA was considered to serve as tumor suppressor gene in gastric cancer by influencing gastric cell-proliferation inhibition and cell death activation^3^. The rs2294008 polymorphism (C to T transition), within the translation starting site of *PSCA*, could lead to a significant reduction of transcriptional activity. Therefore, a decreased PSCA expression associated with the T allele could render an individual more susceptible to develop GC. In contrast to the role of PSCA in gastric cancer, there was evidence demonstrated that PSCA was overexpressed in bladder cancer and the T allele was linked to an increased *PSCA* mRNA expression in both bladder tumor and adjacent normal tissues^10^,^23^. The discrepancy reflects that the regulation and biological function of PSCA is complex and depends on the cell-type context. The study on the expression and function of PSCA in cervical cancer is limited. A study conducted by Liu *et al*.^8^ displayed that PSCA was upregulated in cervical cancer and its expression level was in relation to the invasion of cervical cancer, indicating an oncogenic role in cervical cancer. In this study, we found that the homozygous variant TT genotype was relevant to a decreased risk of cervical cancer (OR = 0.55, 95% CI = 0.38–0.79) in the combined set. Intriguingly, the magnitude of association of the T allele with reduced risk of cervical cancer was comparable with that observed in esophageal adenocarcinoma and squamous cell carcinoma (OR, 0.5; 95% CI: 0.3–0.9 and OR, 0.4; 95% CI: 0.2–0.9, respectively)^19^. Furthermore, the individuals with TT genotype had significantly reduced PSCA protein expression compared with those with CC/CT genotypes. We propose that the variant T allele may result in a decreased transcriptional activity of *PSCA* and thus protein expression in cervical cancer, and consequently conferred reduced cervical cancer susceptibility. Further functional studies characterizing the role of PSCA in cervical cancer and the effect of the rs2294008 polymorphism on PSCA expression are warranted.

Apart from rs2294008, there are other loci at the PSCA gene such as rs2976392 and rs2978974 to be identified in association with cervical cancer susceptibility. In 2008, Sakamoto *et al*.^3^ firstly identified a significant association between rs2976392 locating in the intron of *PSCA* and diffuse-type gastric cancer. Because rs2294008 can modulate transcriptional activity of the upstream region of PSCA and has a strong LD with rs2976392 (*r*^2^ = 0.995, *D*’ = 0.999), they proposed that the functional SNP is the former, rs2294008. After Wu *et al*.^4^ reported a significant association between the rs2294008 and bladder cancer in 2009, Fu *et al*.^23^ performed a fine-mapping study of the 8q24.3, where PSCA maps, by imputing 642 SNPs within 100 kb of rs2294008 in addition to 33 other markers. A multivariable logistic regression model adjusted for rs2294008 revealed a unique signal, rs2978974 (*r*^2^ = 0.02, *D*’ = 0.19 with rs2294008), which locates 10 kb upstream of rs2294008. Although the non-risk allele G of rs2978974 interacted strongly with nuclear proteins which implying a regulatory function, rs2978974 was not associated with *PSCA* mRNA expression. On the contrary, the T risk allele of rs2294008 was associated with increased *PSCA* mRNA expression, suggesting that both variants may be important for bladder cancer susceptibility, possibly through different mechanisms. Fu *et al*.^23^ also found a significant multiplicative interaction between rs2294008 and rs2978974, implying a joint effect of two SNPs on cancer susceptibility. Therefore, the association between rs2978974 joint with or without rs2294008 and cervical cancer susceptibility deserves further investigation.

Some limitations in this study should be addressed. First, the information on human papillomavirus (HPV) infection in subjects is missing. HPV is the necessary cause of cervical cancer^24^. However, HPV is not the necessity in diagnose of cervical cancer (NCCN Clinical Practice Guidelines in Oncology: Cervical Cancer. Version 2.2015). In addition, HPV screening is expensive and requires a handsome amount of money to establish a high-standard testing system with well-trained cytologists who can accurately identify the cells scraped from the cervix, which limiting its wide application in population screening and physical examination in China^25^. Second, the functionality of PSCA in cervical carcinogenesis and the underlying molecular mechanism of rs2294008 modulating cervical cancer susceptibility need further evaluation. Third, we compared the distribution of rs2294008 genotypes between the cases with FFPE tissues and the controls with blood samples in the validation set. Genotyping DNA from FFPE tissues may be inaccurate due to FFPE storage, genetic aberrations, and/or insufficient DNA extraction^26^. Recently, Hertz *et al*. performed a study to investigate the genotyping concordance in DNA extracted from FFPE breast tumor and whole blood, and found that no- and discordant-call rates were below concerning thresholds, and most SNPs could be accurately genotyped from FFPE tissues^26^. In the present study, we compared the distribution of rs2294008 genotypes between the DNA extracted from blood samples (571) and DNA from FFPE tissues (555) using χ^2^ test. The resultant *P* value was 0.882, indicating no significant difference in frequency distribution of rs2294008 genotypes between the two sources of DNA. Finally, there is no IHC staining of PSCA in the controls due to lack of normal tissues adjacent to cervical tumors, which making us unable to compare the IHC staining of PSCA of cervical tumors with that of controls. It is warranted to collect normal tissues adjacent to tumors as much as possible for the future gene expression analyses between the tumors and controls.

In conclusion, the *PSCA* rs2294008 polymorphism was associated with a reduced risk of cervical cancer in a Chinese population. Further studies with large samples at different geographic areas and ethnic groups are warranted to confirm and extend our findings.

## Additional Information

**How to cite this article**: Wang, S. *et al. PSCA* rs2294008 polymorphism contributes to the decreased risk for cervical cancer in a Chinese population. *Sci. Rep.* **6**, 23465; doi: 10.1038/srep23465 (2016).

## Figures and Tables

**Table 1:** Frequency distribution of select variables in cervical cancer cases and controls.

**Variables**^a^	**Test set**		***P***	**Validation set**		***P***
	**Cases**	**Controls**		**Cases**	**Controls**	
	**n** **=** **571 (%)**	**n** **=** **657 (%)**		**n** **=** **555 (%)**	**n** **=** **580 (%)**	
Age, year (mean ± SD)	47.5 ± 10.1	47.3 ± 10.6	0.726	51.3 ± 9.3	51.2 ± 11.0	0.789
Parity						
0–1	324 (58.3)	446 (75.2)	<0.001	297 (54.6)	455 (82.4)	<0.001
≥2	232 (41.7)	147 (24.8)		247 (45.4)	97 (17.6)	
Abortion						
No	158 (29.5)	156 (28.0)	0.565	215 (39.7)	217 (39.5)	0.942
Yes	377 (70.5)	402 (72.0)		326 (60.3)	332 (60.5)	
Menopausal status						
Premenopausal	306 (56.0)	476 (78.9)	<0.001	291 (52.8)	233 (42.6)	<0.001
Postmenopausal	240 (44.0)	127 (21.1)		260 (47.2)	314 (57.4)	
Histologic types						
Squamous cell carcinoma	538 (94.2)			506 (91.2)		
Adenocarcinomas	24 (4.2)			28 (5.1)		
Adenosquamous carcinoma	4 (0.7)			12 (2.2)		
Others^b^	5 (0.9)			9 (1.5)		
Depth of invasion						
I	383 (68)			357 (65.0)		
II	145 (25.8)			189 (34.4)		
III	28 (5.0)			2 (0.4)		
IV	7 (1.2)			1 (0.2)		

^a^Some cases lack information of selected variables.

^b^Other histological types, such as cervical choriocarcinoma.

**Table 2:** Association between *PSCA* SNP rs2294008 and risk of cervical cancer.

**Genotype**	**Combined set**		***P***	**OR (95**% CI)^a^
	**Cases**	**Controls**		
	**n** **=** **1126 (%)**	**n** **=** **1237 (%)**		
CC	609 (54.1)	618 (50.0)	0.002	1.00 (Ref.)
CT	469 (41.6)	527 (42.6)		0.91 (0.77–1.09)
TT	48 (4.3)	92 (7.4)		0.53 (0.37–0.77)
T allele	0.251	0.287	0.005	
CC/CT	1078 (95.7)	1145 (92.6)	0.001	1.00 (Ref.)
TT	48 (4.3)	92 (7.4)		0.55 (0.38–0.79)

^a^Adjusted for age, parity and menopausal status in logistic regression model.

**Table 3:** Stratified analysis of the SNP rs2294008 genotypes associated with cervical cancer risk by selected variables.

**Variables**	**Genotypes (cases/controls)**		***P***	**Adjusted OR (95**% CI)^a^
	**CC/CT**	**TT**		
Set				
Test	545/604	26/53	0.012	0.54 (0.33–0.89)
Validation	533/541	22/39	0.039	0.56 (0.34–0.98)
Age (years)				
≤ 49	584/638	27/44	0.109	0.72 (0.44–1.19)
>49	494/507	21/48	0.002	0.42 (0.25–0.72)
Parity				
0–1	592/834	29/67	0.029	0.63 (0.40–0.99)
≥2	461/228	18/16	0.093	0.57 (0.28–1.14)
Abortion				
No	350/345	23/26	0.643	0.82 (0.46–1.49)
Yes	679/676	24/58	<0.001	0.42 (0.26–0.69)
Menopausal status				
Premenopausal	570/657	27/52	0.034	0.60 (0.37–0.97)
Postmenopausal	479/404	21/37	0.008	0.50 (0.29–0.87)
Depth of invasion				
I	710/1145	30/92		0.53 (0.34–0.81)
II	317/1145	17/92		0.67 (0.39–1.15)
III/IV	37/1145	1/92		0.33 (0.05–2.46)

^a^Adjusted for age, parity, and menopausal status in logistic regression model.
